# Putative Conjugative Plasmids with *tcdB* and *cdtAB* Genes in *Clostridioides difficile*

**DOI:** 10.3201/eid2609.191447

**Published:** 2020-09

**Authors:** Gabriel Ramírez-Vargas, César Rodríguez

**Affiliations:** Facultad de Microbiología and Centro de Investigación en Enfermedades Tropicales, Universidad de Costa Rica, San José, Costa Rica

**Keywords:** bacteria, *C. difficile*, CDT, *Clostridioides*, cytotoxins, lateral gene transfer, mobile genetic elements, plasmids, *tcdB*, virulence factors, *Clostridioides difficile*

## Abstract

The major toxins of *Clostridioides difficile* (TcdA, TcdB, CDT) are chromosomally encoded in nearly all known strains. Following up on previous findings, we identified 5 examples of a family of putative conjugative plasmids with *tcdB* and *cdtAB* in clinical *C. difficile* isolates from multilocus sequence typing clades C-I, 2, and 4.

*Clostridioides difficile* spores may differentiate in the colon of susceptible humans into vegetative cells and release 1 or 2 large clostridial cytotoxins (TcdA, TcdB) or a binary toxin with ADP-ribosyltransferase activity (CDT), or both, to cause colitis and diarrhea (*1*). When present, genes for TcdA, TcdB, and CDT are almost without exception encoded by 2 separate chromosomal loci known as PaLoc and CdtLoc (*2*). Recent discovery of clade C-I strains SA10-050 and CD10-165 in France (*3*) and HSJD-312 and HMX-152 in Costa Rica (*4*) challenged this paradigm, as these strains carry a monotoxin *tcdB*^+^ PaLoc next to a full CdtLoc on extrachromosomal molecules that resemble conjugative plasmids (*4*). 

The Anaerobic Bacteriology Research Laboratory (LIBA) has been isolating and typing *C. difficile* in Costa Rica for nearly a decade and thereby generated an isolate collection with >800 records. We searched mobile genetic elements (MGEs) among whole-genome sequences from 150 of those bacteria, leading to the discovery of 5 new *tcdA*^–^/*tcdB*^+^/*cdtAB*^+^ putative plasmids among isolates that were cultivated from loose fecal samples of patients under clinical suspicion for *C. difficile* infections (CDIs): LIBA-6656, LIBA-7194, LIBA-7602, LIBA-7678, and LIBA-7697. These materials were collected at 3 hospitals located within a 78.5 km^2^ area in 2013 (LIBA-6656), 2016 (LIBA-7194), 2017 (LIBA-7602), and 2018 (LIBA-7678, LIBA-7697). Raw sequencing data can be retrieved from the European Nucleotide Archive (https://www.ebi.ac.uk/ena; LIBA-6656, run ERR467623) or from the MicrobesNG platform (https://microbesng.com/portal/projects/FB43968C-E9EF-4270-9D1A-054457CC9B54). 

A tree of aligned, concatenated, multilocus sequence typing allele combinations revealed that the new plasmid sequences were present in isolates assigned to clade C-I (LIBA-7194, LIBA-7602, LIBA-7678), clade 2 (LIBA-6656), and clade 4 (LIBA-7697) (Figure 1, panel A). This unexpected result expands the host range of this type of MGE to include *C. difficile* clades more commonly associated with human hosts. The 3 clade C-I strains were different, as confirmed by pairwise estimates of genomic MinHash (min-wise independent permutations locality sensitive hashing scheme) distances (0.022–0.049) calculated with MASH (https://mash.readthedocs.io/en/latest/index.html) and average nucleotide identities (94.15%–97.45%) calculated with FastANI (https://github.com/ParBLiSS/FastANI). Genome similarity showed a tendency to decrease with time (data not shown), suggesting that clade C-I strains are evolving. 

Although our plasmid assemblies are awaiting confirmation by long-read sequencing, the size of 3 of the reconstructed plasmids (139.2–147.7 kb) closely matches that of known *C. difficile* toxin plasmids, such as pHSJD-312 (145.1 kb) (*4*). The toxin contigs of LIBA-7697 (53.2 kb) and LIBA-7194 (228.8 kb) were fragmented or likely misassembled, respectively. Read mapping to a high-quality hybrid assembly of pHSJD-312 showed 53.6%–93.7% identical sites in the alignment and 92%–98% reference sequence coverage, indicating that the new toxin plasmids are not the same molecule (Figure 1, panel B). We corroborated this result with a Panaroo (https://github.com/gtonkinhill/panaroo) pangenome analysis of pHSJD-312 and the plasmid sequences found in LIBA-6656, LIBA-7602, and LIBA-7678, because it classified only 135 (89%) of 152 genes as conserved. This core genome included toxin loci, agr loci, and potential conjugation systems. In contrast, mapping gaps corresponded to putative virulence factors (i.e., lectin-binding or cell wall–binding proteins), hypothetical proteins, and MGEs, such as class 2 introns and transposases (Figure 1, panel B). These findings imply that this group of chimeric molecules is undergoing nonhomologous recombination. 

The MGE-associated *tcdB* sequence of LIBA-6656 (clade 2) could not be fully assembled. In the remaining 4 strains, this gene was highly conserved (99%–100% protein sequence identity) and expected to encode variant TcdBs that would cause a *Clostridium sordellii*–like cytopathic effect. Besides its plasmid-borne *tcdB*, LIBA-6656 carries a different *tcdB* allele on a chromosomal PaLoc. The contribution of each of these *tcdB* alleles to infection is unclear at this time. Yet, the coexistence of 2 PaLocs within a host is compatible with the suggested transition from ancient monotoxin PaLocs to modern bitoxin PaLocs (*3*). We also noted a high level of sequence identity for *cdtA* (≥99%) and *cdtB* (≥98%) in all 5 putative plasmids. However, it is difficult with such a small dataset to conclude whether the noted conservation of toxin gene sequences reflects stable coevolution or only the short evolutionary time after acquisition. 

As previously seen in other clade C-I toxin plasmids, the toxin genes of the new putative plasmids are flanked by genes for a transposase and an integrase (*4*). Furthermore, we identified their PaLocs as lateral gene transfer events using Alien_Hunter software (Sanger Institute, https://www.sanger.ac.uk). Additional elements from this group of MGEs lack toxin genes (*4*), indicating that they are gained through lateral gene transfer.

Three of the 5 isolates that host new toxin plasmids would have remained undetected if we had not attempted *C. difficile* cultivation from TcdB^–^ fecal samples or sequencing for isolates with negative results for *tcdC* and *tcdA* (LIBA-7194, LIBA-7602, LIBA-7678). We therefore anticipate that the frequency of *C. difficile* isolates with toxin plasmids has been underestimated and recommend that current diagnostic procedures be refined. Moreover, our results open avenues to explore whether similar plasmids are present in species other than *C. difficile* and are implicated in undiagnosed cases of antibiotic-associated diarrhea. 

**Figure:**
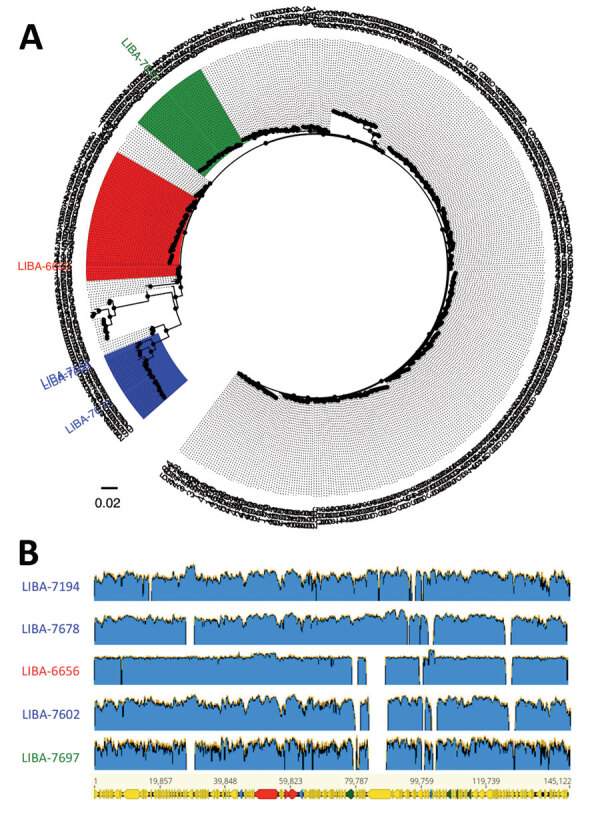
Multilocus sequence typing–based classification (A) and diversity of extrachromosomal circular sequences (B) of *Clostridioides difficile* strains with plasmid-encoded toxins. A) FastTree (http://www.microbesonline.org/fasttree) phylogenic tree derived from a MUSCLE (http://www.drive5.com/muscle) alignment of concatenated multilocus sequence typing alleles from all *C. difficile* sequence types deposited in the PubMLST database (https://pubmlst.org). Tip labels represent sequence types or strain names. Strains from clade C-I are highlighted in blue, from clade 2 in red, and from clade 4 in green. B) This graphic shows short reads from strains LIBA-7194, LIBA-7678, LIBA-7602 (clade C-I, blue), LIBA-6656 (clade 2, red), and LIBA-7697 (clade 4, green) mapped to the plasmid sequence of strain HSJD-312, which was obtained through hybrid PacBio (Pacific Biosciences, https://www.pacb.com) and Illumina (Illumina, https://www.illumina.com) sequencing and therefore used as a reference (145.1 kb, bottom). Arrows in the reference sequence represent annotated coding sequences. Genes for toxins are in red; for transposases, integrases, and recombinases are in blue, and for proteins from a putative conjugation machinery are in green.

## References

[1] Balsells E, Shi T, Leese C, Lyell I, Burrows J, Wiuff C, et al. Global burden of *Clostridium difficile* infections: a systematic review and meta-analysis. J Glob Health. 2019;9:010407. 10.7189/jogh.09.010407 30603078 PMC6304170

[2] Knight DR, Elliott B, Chang BJ, Perkins TT, Riley TV. Diversity and evolution in the genome of *Clostridium difficile.* Clin Microbiol Rev. 2015;28:721–41. 10.1128/CMR.00127-14 26085550 PMC4475645

[3] Monot M, Eckert C, Lemire A, Hamiot A, Dubois T, Tessier C, et al. *Clostridium difficile*: new insights into the evolution of the pathogenicity locus. Sci Rep. 2015;5:15023. 10.1038/srep15023 26446480 PMC4597214

[4] Ramírez-Vargas G, López-Ureña D, Badilla A, Orozco-Aguilar J, Murillo T, Rojas P, et al. Novel Clade C-I *Clostridium difficile* strains escape diagnostic tests, differ in pathogenicity potential and carry toxins on extrachromosomal elements. Sci Rep. 2018;8:13951. 10.1038/s41598-018-32390-6 30224751 PMC6141592

